# The Role of Lower Airway Dysbiosis in Asthma: Dysbiosis and Asthma

**DOI:** 10.1155/2017/3890601

**Published:** 2017-12-13

**Authors:** Junying Lu, Lingxin Xiong, Xiaohao Zhang, Zhongmin Liu, Shiji Wang, Chao Zhang, Jingtong Zheng, Guoqiang Wang, Ruipeng Zheng, Jodie L. Simpson, Fang Wang

**Affiliations:** ^1^Department of Pathogeny Biology, College of Basic Medical Sciences, Jilin University, Changchun 130021, China; ^2^Department of Intensive Care Unit, First Hospital of Jilin University, Changchun 130021, China; ^3^School of Pharmaceutical Sciences, Jilin University, Changchun 130021, China; ^4^Department of Cardiology, Second Hospital of Jilin University, Changchun 130041, China; ^5^Department of Interventional Therapy, First Hospital of Jilin University, Changchun 130021, China; ^6^Department of Respiratory and Sleep Medicine, University of Newcastle, New Lambton, NSW, Australia

## Abstract

With the development of culture-independent techniques, numerous studies have demonstrated that the lower airway is not sterile in health and harbors diverse microbial communities. Furthermore, new evidence suggests that there is a distinct lower airway microbiome in those with chronic respiratory disease. To understand the role of lower airway dysbiosis in the pathogenesis of asthma, in this article, we review the published reports about the lung microbiome of healthy controls, provide an outlook on the contribution of lower airway dysbiosis to asthma, especially steroid-resistant asthma, and discuss the potential therapies targeted for lower airway dysbiosis.

## 1. Introduction

The human body is colonized by trillions of commensal microorganisms known as the human microbiome covering the skin, intestinal tract, the upper respiratory tract, and oral cavity, as well as genitourinary tract [1]. These microorganisms may be beneficial or potentially pathogenic. The human microbiome constitutes a complex ecosystem in which there is a generally symbiotic interaction between the host and microorganisms. The maintenance of a harmonious state between microorganisms and host accounts for many beneficial effects. For example, the intestinal microbiome is known to influence host immunity by balancing the activities of T helper (Th) type 1 and Th-2 cells and playing a fundamental role in the induction of inflammatory or tolerogenic conditions [2, 3]. Dysbiosis, known as the imbalance in the composition of the bacterial microbiome, relates with host inflammatory response [4, 5] may also be an important major factor in the development or the chronicity of human disorders such as inflammatory bowel disease, diabetes, and asthma [6–8].

In the last six years, with the development of sequencing technologies, the lower airway is no longer considered to be sterile and a growing body of evidence supports the concept that the healthy lung microbiome may have transient changes that may impact the progression of the disease [7, 9–13]. Furthermore, new evidence suggests that there are differences in microbiome in lower airways between the healthy subjects and those with chronic respiratory diseases such as chronic obstructive pulmonary disease (COPD) and asthma [7, 14–16].

Asthma is a heterogeneous inflammatory disease, manifesting in recurrent exacerbations of wheeze, dyspnea, chest tightness, and cough, with many distinct phenotypes and endotypes [17, 18]. The symptoms of most patients with asthma can be relieved by short-acting beta2 agonists and low doses of inhaled corticosteroids (ICS) which is the first-line treatment for the majority of patients; however, approximately 10% of patients requires the maximal ICS dose [19], and between 5% and 10% of adults with asthma are resistant to steroid treatment, accounting for over one-half of asthma-related healthcare costs [20]. Understanding the contribution of lower airway microbiota to the pathogenesis of asthma, especially steroid-resistant asthma, could transform our understanding of asthma and provide novel therapeutic targets. To understand the role of lower airway dysbiosis in the pathogenesis of asthma, in this article, we review the published reports about the lung microbiome in both healthy subjects and those with stable asthma in the absence of infection. The aim of the review is to consolidate current knowledge of the airway microbiome in healthy controls, to provide an outlook on both airway dysbiosis and the altered host immune system, and to discuss the potential therapies targeted for lower airway dysbiosis.

## 2. New Challenges: The Lung Microbiome in Health

The human microbiome has the extraordinary capability to exist even in a hostile environment. This includes the lower airway, due to its continual exposure to the environment, with high volume airflow through the damp upper airway which harbors a complex microbial population [21]. High-throughput next-generation sequencing techniques have reported that similar to other epithelial surfaces, the lower airways harbor diverse microbial communities [10–13, 22–24] which exert an effect on the local immunological homeostasis in normal individuals [4, 5].

### 2.1. Lower Airway Microbiome in Healthy Subjects

Hilty and colleagues first described that the lower airway in eight healthy subjects contained a characteristic microbiome using 16S rRNA gene marker sequencing technology from bronchoscopy-derived samples in 2010, and the bacteria are distinct from those from the upper respiratory tract [10], while Charlson et al. found a lung-specific microbiome that resembled the upper airway in six healthy individuals by measuring bacterial abundance and composition at multiple sites by the 16S rDNA gene marker [11]. Since then, numerous studies have described the characterization of the lower airway microbiome in healthy subjects, comprising of a low bacterial load with a relatively diverse bacterial composition [11–13, 22–24]. When analyzed at the phylum level, Proteobacteria, Firmicutes, and Bacteroidetes are the dominant phyla observed in the lower airways of the healthy. The predominant bacterial genus among healthy subjects includes *Streptococcus, Pseudomonas*, *Prevotella*, *Fusobacteria*, *Veillonella*, *Neisseria*, and *Haemophilus* [10, 12, 23–25].

Interestingly, a core group of pulmonary bacteria has been suggested in healthy subjects. Bronchoalveolar lavage (BAL) samples from ten healthy subjects identified seven core members which included *Pseudomonas*, *Streptococcus*, *Prevotella*, *Fusobacterium*, *Haemophilus*, *Veillonella*, and *Porphyromonas* [12]. Another study of 257 sputum samples identified four key members including *Prevotella*, *Neisseria*, *Veillonella*, and *Streptococcus* [24]. However, a murine study found that the predominant bacterial shifts from Gammaproteobacteria and Firmicutes towards Bacteroidetes at phyla level during the first 2 weeks after birth [26]. Meantime, a study of healthy infants showed that the respiratory microbiota is dynamic [27]. Therefore, whether there is a permanent core group it needs further research.

As the study on the lung microbiome is a new field, there is not enough studies that have looked at this question and requires further clarification; it is necessary to standardize the technology and analysis to allow comparison of the growing data to build our understanding of the composition of the healthy airway microbiome.

### 2.2. Factors Which Impact the Composition of the Lower Airway Microbiome

Evidence suggests that the lower airway microbiota might be associated with host factors such as genetic polymorphism and immune status as well as multiple environmental factors (e.g., environmental exposures, and origin, and lifestyle), as it is the case for the intestinal microbiota [24, 25].

Host genetics was found to be a significant factor which influences the composition of the airway microbiota in a twin-family cohort study by estimating the heritability of each microbial taxon [24]. A study of a twin-family cohort demonstrated that host genetic polymorphism can affect the composition of the airway microbiome [24]. 16S rRNA-based analysis of the gut microbiome in 1126 twin pairs from the United Kingdom revealed that certain species containing Bifidobacterium was heritable and fabricable. Like the gut microbiome, there may be genetic factors in the origins of lower airway microbiome [28]. In addition, fetal lungs probably acquire their microbial communities after birth. As a fetus transits out through the birth canal, the infant mucosal surfaces are covered with their mother's microbes which differentiate into site-specific communities in subsequent days and weeks [29, 30]. Alternately, host immune status may also impact on the airway microbiota. An examination of the lung microbiome in HIV-infected patients found that *Tropheryma whipplei* was highly more frequent in the BAL of HIV-positive individuals compared with HIV-negative individuals [31]. This result was consistent with another study in which the alteration of lung microbiome was reported to be associated with HIV infection [32]. And a new study published by Moreira and colleagues shows how the bacterium *Burkholderia multivorans* evolves and adapts in the lungs of cystic fibrosis patients [33]. The above results show that the airway microbiome is influenced by the host immune response, and the bacteria may evolve in adapting to a specific lung environment.

The microbiome is also influenced by its environment. In early life, children who grow up on a farm or have close contact with farm animals and pet dogs have a reduced risk of asthma, which is thought to be due to increased exposure to a diverse range of bacteria [34–37]. Lynch and colleagues used culture-independent techniques to examine the bacterial composition of house dust samples collected during the first year of life. The results demonstrated that healthy children were exposed to richer and more diverse bacterial communities compared to the children who developed either atopy or reported recurrent wheeze [38]. Geographical variance should be also considered when interpreting microbiota data as pronounced differences in the intestinal microbiome according to different geographic origins have been reported [39], and different airway microbiota have been observed in cystic fibrosis patients coming from different countries [40]. The impact of the composition of aerial bacterial communities and different geographical origins on the constitution of lower airway microbiota has not been described in previous studies. Among the multiple lifestyle factors, cigarette smoking and airway inflammation appear to alter the composition of the microbiome from the upper airways (including nasopharynx and oropharynx) in a study of healthy asymptomatic adults including 29 smokers and 33 nonsmokers [41]. Additionally, another study on 257 sputum samples from healthy adults showed that smoking status was significantly correlated with the microbial community structure, and 54 current smokers were significantly enriched in the genera *Veillonella* and *Megasphaera* compared with 170 nonsmokers. This suggests that cigarette smoking can influence the overall structure of the microbial community [24]. Conversely, other studies show a similar heterogeneity of the lower airway bacterial communities which exist in healthy smokers and nonsmokers [12, 23]. Additionally, a study examining induced sputum samples from 44 asthmatic smokers demonstrated that there was greater bacterial diversity in smokers with asthma compared with nonsmoking healthy controls, and that smoking cessation did not lead to a change in the bacterial communities [42]. Currently, our understanding of the effect of cigarette smoke on the lower airway is very poor, more attention and focus should be required to understand tobacco smoke's effect on the lower airway microbiome.

In summary, both host and environmental factors may contribute to the composition of the lower airway microbiome.

### 2.3. The Origins of the Lower Airway Microbiome

Microbial colonization begins immediately after birth. As the fetus is delivered vaginally or via Cesarean section, the infant mucosal surfaces are covered with their mother's microbes which are different according to their delivery mode and differentiate into site-specific communities in subsequent days and weeks, so the fetal lungs likely acquire their microbial communities shortly after birth [29, 30]. More recently, studies showed that the respiratory microbiota of healthy infants are gradually developing into dynamic and complex microbial communities which may influence the susceptibility to respiratory diseases [27, 43]. However, most airway microbiota studies in healthy infants are based on the upper airways due to the practicality in obtaining samples from that population.

In adults, the lower airway microbiome is likely to be populated via the upper airway (nose and throat), mouth, and gastrointestinal tract. Charlson et al. performed intensive sampling of multiple sites along the respiratory tract in six healthy individuals using a two-bronchoscope technique to avoid cross-contamination of samples and described community composition and diversity of the entire respiratory tract microbiome [11]. The study showed that the respiratory tract microbiome was homogenous with a lower bacterial load in the lower airway and indistinguishable in community composition compared with the upper airway. Conversely, a study examining the composition of the lower respiratory tract microbiome in 10 healthy participants found that the lung bacterial communities resembled those from the mouth but identified a specific lung microbiome with higher abundance, demonstrating that the lung microbiome did not derive solely from the mouth [44]. This result is similar with a recent finding showing that the main source of the healthy lung microbiome is the mouth, which is caused by microaspiration while the nose makes little contribution [45].

## 3. Lower Airway Dysbiosis and Asthma

Since the 1980s, a marked increase in the prevalence of atopic diseases has been steadily increasing in developed countries. Furthermore, the increase in atopic diseases is accompanied by a reduction of infectious agents in the same time period. Strachan proposed the well-known “hygiene hypothesis” which suggests that the lack of exposure to the microbiome may contribute to the increased prevalence of atopic diseases, and since this time, the association between the microbiome and asthma has been studied [46]. Several epidemiological studies have shown that exposure to a diverse microbial environment in early life was inversely related with the risk of asthma [34, 36, 47]. Previous studies on the microbiome in asthma have mainly focused on intestinal microbial communities and their roles in the development of immune function through the concept of a “common mucosal response” [48, 49]. Recently, with the understanding of the microbiome in the lower respiratory tract, more and more studies are now focusing on the relationship between colonization of the lower airway microbiome and immune tolerance as well as the impact of lower airway dysbiosis on chronic airway diseases including asthma.

### 3.1. Lower Airway Dysbiosis in Adult Asthma

Sampling methods of the lower airways are typically based on BAL, bronchial brushes, or collection of induced sputum [50]. Despite differences in sampling technique, evidence suggests that airway dysbiosis, characterized by an altered composition of the microbiome and dominant flora, may play a prominent role in the asthmatic airways compared to healthy subjects [7, 10, 44, 51]. Characterization of the airway dysbiosis in asthma could be beneficial to assess the impact of antimicrobial therapy and identify new treatment strategies.

Hilty and colleagues first suggested that there may be a disturbed composition of the microbiome in the asthmatic lower airways where a significant increase in Proteobacteria (especially *Haemophilus spp.*) was found in the BAL of 11 adults and 13 children with asthma, most of whom were treated with ICS. In contrast, Bacteroidetes (specifically *Prevotella spp*.) were more common in controls than in asthma [10]. Other studies have demonstrated a similar result with more Proteobacteria found in those with asthma compared with healthy controls. One study assessed 10 patients with mild active asthma (8/10 were not using ICS) sampled using induced sputum, and another study collected bronchial epithelial brushing from 65 suboptimally controlled asthma most of whom were taking ICS [44, 51]. The patients in the above studies had mild-to-moderate asthma. In patients with severe asthma, the lower airway microbiome of bronchial brushing from 40 participants most of whom were refractory to corticosteroids was significantly enriched in Actinobacteria compared with mild asthma [7].

Diversity of the microbiome is important to promote host immunity and may become a new biomarker of health [52]. Previous studies have reported increased diversity of opportunistic pathogens and decreased diversity of beneficial flora in asthma compared with healthy controls and that increased diversity was significantly correlated with bronchial hyperresponsiveness [44, 51]. Moreover, other investigations showed a similar microbiota diversity between asthma and controls [10, 53]. Loss of microbiota diversity is another manifestation of microbiome dysbiosis. When patients with asthma were further characterized according to their inflammatory subtypes, 7 participants with poorly controlled neutrophilic asthma, the majority of whom were taking high-dose ICS, had reduced bacterial diversity in induced sputum samples compared with other asthma inflammatory subtypes [16]. Interestingly, in patients with COPD, a similar discrepancy where increased or decreased diversity of lower airway microbiota is reported [12, 54]. As described in the previous section, the composition of the airway microbiome can be influenced by multiple factors including host state and environment, which may explain the observed inconsistencies within the current data, but whether the difference is associated with the pathogenesis of asthma remains unclear and needs further investigation.

### 3.2. Lower Airway Dysbiosis and the Features of Adult Asthma

Airway dysbiosis is associated with several disease-related features of asthma [7, 14, 51]. Studies showed bacterial diversity and composition of the airway were significantly associated with the severity of airflow obstruction [14] and bronchial hyperresponsiveness [51] and that the abundance of Proteobacteria was associated with worsening asthma control questionnaire (ACQ) scores and positively correlated with Th-17 associated gene expression [7]. Thus, specific Proteobacteria families may play a role in Th-17 associated neutrophilic asthma, in agreement with our previous report of a high abundance of *Haemophilus* sequences in patients with neutrophilic asthma [16]. Airway dysbiosis is associated with increased gene markers, including steroid responsiveness FKBP5 and TH-17 phenotype, representing an activation of innate immune pathways that may influence the phenotype and severity of asthma [7]. Association between specific predominant microbes and a typical airway inflammatory phenotype provides a strong evidence for investigating the role of airway dysbiosis in asthma.

### 3.3. Airway Dysbiosis as a Cause of Asthma?

The selective pressure of the host immune response may result in a distinct microbiota pattern of the lung [31]. It is yet unclear whether airway dysbiosis is the cause or consequence of airway inflammation in asthmatic patients, animal models may give some insights; however, it is important to interpret results with caution as they need to be confirmed in clinical studies.

Studies of germ-free and neonatal mice demonstrate a direct contribution of the microbiome establishment in early life on the severity and development of allergic airway inflammation [26, 55]. When exposed to ovalbumin (OVA), germ-free mice were prone to develop allergic airway inflammation, characterized by exaggerated airway eosinophilia and increased Th-2 cell cytokines and airway hyperresponsiveness, suggesting that the presence of commensal bacteria is critical for the homeostasis of allergic airway inflammation [55]. Recently, the lower respiratory microbiome in a murine model of allergic airway inflammation was shown to play an important role in the development of allergic immune response following exposure to house dust mite (HDM) [26]. During the first two-weeks after birth, there was an increased bacterial load and changes in bacterial community composition in the lungs which was associated with decreased allergen responsiveness and lower airways eosinophilia and decreased Th-2 cytokine (including IL-4, IL-5, and IL-13) production in BALF. In agreement with these finding, Schuijs et al. demonstrated that persistent exposure to farm dust and endotoxin protected mice from HDM-induced asthma by reducing airway epithelial cell cytokines which activate dendritic cells, thus suppressing Th-2 immune response to HDM allergen [56]. Therefore, the lack of bacterial colonization after birth resulted in exaggerated allergic reactions which persisted into adulthood, indicating that the lung microbiome is dynamic and airway dysbiosis in early life can result in sustained susceptibility to airway allergic diseases [26, 56]. Like the role of the intestinal microbiome, the lower airway microbiome may contribute to maintaining local homeostatic and inflammatory immune responses of the lung [57, 58]. However, asthma is a heterogeneous disease, and there is a complex interaction between the lung microbiome and host immunity which may impact on the risk of asthma development. The role of airway dysbiosis in the development of asthma needs further study.

## 4. Corticosteroids and Airway Dysbiosis

### 4.1. Do Corticosteroids Induce the Airway Dysbiosis in Asthma?

Current guidelines recommend the use of ICS for asthma to control airway inflammation [17]. The impact of corticosteroids on the airway microbiome is unclear. Clinical studies have reported that ICS therapy is significantly associated with an increased risk of pneumonia [59, 60]. In a mouse model of acute respiratory infection, glucocorticosteroids promoted *Haemophilus influenza* persistent colonization [61], suggesting that the presence of corticosteroids can promote colonization of potentially pathogenic bacteria. Do corticosteroids induce the airway dysbiosis in asthma?

In mild-to-moderate asthma, Marri et al. showed that 10 patients with mild asthma, most of whom were not taking ICS, contained higher proportions of Proteobacteria in sputum samples [44]. This finding is consistent with another study in which all 11 patients with mild-moderate asthma treated with ICS found that pathogenic Proteobacteria, especially *Haemophilus spp.*, was more frequently found in the bronchi of adult and children asthmatic patients than controls [10], and a study by Huang et al. who found Proteobacteria in bronchial epithelial brushing from 65 adults with suboptimally controlled asthma took ICS for at least 4 weeks. In summary, Proteobacteria is the dominant species in the patients with mild-moderate asthma, irrespective of the use of ICS, suggesting that airway dysbiosis may be a feature of asthma itself and is not solely attributable to ICS therapy. In fact, a recent study confirmed that the diversity and composition of the bronchial airway microbiome was not influenced by ICS treatment alone but influenced by the combination therapy of oral and ICS treatments [14].

### 4.2. Does Airway Dysbiosis Contribute to Steroid Resistance in Asthma

While ICS treatment may not result in airway dysbiosis, it is unclear whether the presence of airway dysbiosis will have an impact on steroid resistance in asthmatic patients.

In a study in 28 treatment-resistant adults with asthma, the relative abundance of *M. catarrhalis*, *Haemophilus*, and *Streptococcus* was associated with longer asthma disease duration, worse postbronchodilator FEV1 percent predicted, and higher sputum neutrophil counts and IL-8 concentrations [62]. Another study comparing the composition of the airway microbiome between corticosteroid-resistant (CR) and corticosteroid-sensitive (CS) patients identified differences at a genus level, with distinct genus expansions in 29 CR and 10 CS patients. The microorganisms expanded in the BAL of those with CR asthma were mainly gram-negative lipopolysaccharide- (LPS-) producing bacteria with known high-endotoxic activity such as *Haemophilus*, *Neisseria*, and *Campylobacter*, while the microbial organisms uniquely expanded in CS asthma showed low endotoxic activity, for example, *Bradyrhizobium* and *Limnobacter* [53]. To examine the effects of the microbiome that was uniquely expanded in CR asthma, human airway macrophages were cocultured with clinical strains of *Haemophilus parainfluenzae* and found to respond with a reduced cellular reaction to corticosteroid with the activation of IL-8 and p38 MAPK [53]. In contrast, inhibition of TAK1, rather than p38 MAPK itself, in MAPK signaling pathway resulted in corticosteroid sensitivity, suggesting corticosteroid resistance was induced by activating TAK1/MAPK, which was triggered by airway expansion of specific gram-negative bacteria. Moreover, an animal study also supported these findings that dose-dependent inhalation of LPS can modulate different asthmatic phenotypes via stimulation of immune responses of the host [63]. In a mouse model of allergic airway inflammation challenged with OVA, an extremely low dose of LPS exposure promoted Th-2 dominant inflammation, whereas moderate dose of LPS promoted Th-17 associated neutrophilic inflammation, and high dose of LPS triggered the accumulation of large numbers of Treg cells [63]. Therefore, airway dysbiosis characterized by an abundance of a specific bacteria may stimulate airway cells via exposure to bacterial LPS, which induces elevated Th-17 response and IL-8 level and recruits neutrophils into the airway lumen.

## 5. New Potential Targeted Therapies for Lower Airway Dysbiosis

Current therapeutic interventions for asthma are mainly restricted to bronchodilators and anti-inflammatory drugs like corticosteroids. Understanding the lower airway microbiota in health and dysbiosis in asthma may open new therapeutic interventions for asthma. These interventions include the use of probiotics or antibiotics attempting to modify the composition of the airway microbiota.

### 5.1. Antibiotic Therapy

#### 5.1.1. Antibiotics and Asthma

The current international guidelines do not support the use of antibiotics for asthma [17]. As studies have shown that microbiomes are closely related with asthma [64] and that patients with asthma have an increased susceptibility to respiratory bacterial infections [65], antibiotics are still frequently prescribed as a general treatment for asthma [66]. However, more and more studies suggest that exposure to antibiotics is associated with the increased risk of subsequent asthma [67–71].

A prospective study showed that exposure to antibiotics in pregnancy increases the risk of asthma exacerbations by twofold in 5-year-old children [67]. Cohort analyses showed positive associations between the increased risk of subsequent asthma and exposure to antibiotics in fetal life especially used for respiratory infections rather than for either urinary tract or skin infections [68]. The use of antibiotics during the first year of life is also believed to increase asthma risk by two- or threefold [69]. In a longitudinal analysis from birth to the age of 11 years, antibiotic prescription increases the risk of subsequent asthma exacerbations and hospital admissions in children with wheezing [70]. However, these associations disappeared in sibling control analyses [68]. Another study with a fairly small study population reported no significant associations between them [71]. Overall, more studies suggest that exposure to antibiotics increase the risk of asthma in fetal life and early childhood. Recently, an animal study has found that intranasal exposure to an antibiotic did alter the lung microbiome [72]. Whether the exposure to antibiotics by acting on lower respiratory tract microbiome has an effect on the risk of asthma requires further study.

#### 5.1.2. Macrolides Antibiotics

Macrolides are common antibiotics in the treatment of both acute and chronic respiratory infections and in those patients who have an allergy to penicillin [73]. The Global Initiative for Asthma (GINA) report recommends that antibiotics should not be routinely used for asthma exacerbations unless there is strong evidence of lung infection (e.g., fever or purulent sputum or radiographic evidence of pneumonia) [17]. Macrolides exert antibacterial properties by inhibiting bacterial protein synthesis by interaction with 50S subunit in the 70S prokaryotic ribosome, and some macrolide antibiotics including azithromycin and clarithromycin share similar interactions with the 23S ribosomal RNA (rRNA) found in the 50S subunit, stimulating the dissociation of peptidyl-tRNA from bacterial ribosome [74, 75]. It was believed that macrolides inhibit synthesis of many proteins by obstructing the egress of nascent proteins. Recently, studies showed that translation of the majority of polypeptides is interrupted by the drug-bound ribosome which becomes arrested at specific, well-defined mRNA sites. Ribosome profiling analysis carried out in *Staphylococcus aureus* and *E. coli* treated with macrolides showed that the major sites of translation arrest are enriched in proline and charged residues [76, 77]. Unlike other macrolides such as telithromycin and clarithromycin, the presence of nitrogen within the lactone ring structure of azithromycin grants resistance to pH changes in phagocytic vesicles, which facilitates delivery and concentration sustaining of azithromycin at sites of infection by host chemotaxis and contributes to antibacterial property by host macrophages [78]. In addition to the direct antibacterial activities, macrolide antibiotics inhibit the production of virulence factors, adherence, motility, and biofilm formation [74, 79, 80]. Recently Segal et al. found that azithromycin may contribute to its anti-inflammatory effects through affecting the metabolism of the lung bacterial [81]. Clarithromycin can reduce Th-2 and TNF-*α* responses in steroid-sensitive allergic airway disease, indicative of a potential widespread applicability in the treatment of asthma [20]. The immunomodulatory effect of clarithromycin was further confirmed by asthma models *in vivo*, in which decreased Th-2 responses including IL-5, IL-13, and eosinophils were observed [20]. In a similar manner, an observational study found consistent with prior reports that the use of clarithromycin 500 mg twice daily reduced sputum IL-8 and downregulation of airway neutrophils [82]. In a large number of manuscripts, macrolides are reported to possess immunomodulatory effects by accumulation within inflammatory cells by the modulation of the ERK1/2 phosphorylation and suppression of NF-*κ*Β activation [75]. Macrolides including clarithromycin and roxithromycin improve clinical features of asthma such as lung function [83, 84] and can reduce the degree of bronchial hyperresponsiveness [83], whereas others have shown an association between macrolide use (six weeks of treatment with roxithromycin 150 mg twice a day), an improvement in clinical symptoms, asthma control, and evening peak expiratory flow [84].

It is possible that the effectiveness of macrolides may be restricted to a specific asthma phenotype or endotype. A review showed that severe, steroid-insensitive, neutrophilic asthma was more responsive to macrolides compared with mild-to-moderate asthma [74]. Similarly, although low doses (250 mg per day for 5 days) of azithromycin did not reduce the frequency of exacerbations and lower respiratory tract infection in adult participants with severe asthma, the significant reduction in primary endpoints in azithromycin-treated patients with noneosinophilic asthma was observed, suggesting a potent role for azithromycin for prevention of noneosinophilic severe asthma [85]. The combination of 16S rRNA gene maker sequencing and macrolides may contribute to individualized therapies based on a distinct diagnosis of phenotypes and endotypes. Analysis of the lung microbiome by 16S rRNA gene maker may decipher whether the beneficial effects of macrolides in the prevention of exacerbations are anti-inflammatory or antibiotic in nature. Furthermore, longitudinal studies are needed to further understand how changes in microbial composition and microbiota structure determine the onset and development of asthma. Identification of these dynamic changes may be beneficial as both a biomarker and a potential therapeutic target.

### 5.2. Probiotics

As discussed above, microbial factors have an important role in the homeostasis of host immune responses and can influence the risk of developing allergic disease in susceptible individuals. One potential way to correct dysbiosis and restore “healthy” lung microbiota may be via supplementation with probiotics or microbiota-derived products by oral administration or inhalation.

Probiotics are live microorganisms, for example, lactic acid bacterial species, which have shown to be beneficial in inflammatory bowel disease by changing the composition of the intestinal microbiota and restoring the microbial intestinal balance [86]. From the 1990s, there were numerous studies designed to evaluate the effects of probiotics treatment or prevention of allergic diseases including atopic dermatitis and allergic rhinitis [87, 88]. Clinical trials have shown that several probiotics showed preventive and therapeutic efficacy against allergic diseases including eczema and allergic rhinitis; nevertheless, studies on the role of probiotics in the administration of asthma remain rare [89, 90].

Evidence from animal studies suggests that oral probiotics in early life can suppress allergic airway inflammation [91–93]. However, clinical studies show inconsistent results; studies of children with asthma show that probiotics have a beneficial effect in improving clinical symptoms and lung function [94, 95], while studies of oral probiotic use in adults and preschool-aged children show no benefit in lung function and bronchial inflammation [96, 97]. There is insufficient evidence to support the use of oral probiotics for the treatment of asthma, and no studies have investigated whether probiotics are able to prevent exacerbations of asthma.

As we learn more about the lower airway microbiome in respiratory disease, it is interesting to question whether oral probiotics can alter and influence the composition of the microbiome in respiratory tract and subsequently airway disease severity, chronicity, or response to therapy. A recent animal study of lung inflammation generated by exposure to carbon nanotube particles in adult mice showed that oral probiotics did not change the lung microbiome [72]. In a mouse model of allergic airway inflammation induced by OVA challenge, pulmonary exposure to an innocuous strain of *Escherichia coli* by inhalation resulted in the suppression of allergic airway responses mainly characterized by significant fewer total cells and lower eosinophil frequency in BAL in *E. coli*-treated mice which suggests bacterial-induced protection against allergic airway inflammation [98]. Further studies to evaluate the applicability of airway exposure by inhalation of microbiota-derived products or probiotics to the composition of the airway microbiome and asthma are needed.

## 6. Summary

With the development of sequencing technology, there are increasing studies that support the hypothesis that the lower airway is not sterile in health and harbors diverse microbial communities of a low bacterial load which can be affected by the host and environmental factors. More and more evidence showed that diverse microbial exposure in early life may modulate host immune response and influence the susceptibility of atopic disease. Asthma is a complex heterogeneous disease. The lower airway dysbiosis has been shown to contribute to the pathophysiology of asthma, especially neutrophilic asthma. The possible mechanism is that an abundance of a specific bacteria may induce elevated Th-17 response via exposure to bacterial LPS. With the understanding of the role of lower airway dysbiosis in the pathogenesis of asthma, the therapeutic interventions targeted for correcting dysbiosis and restoring “healthy” lung microbiota may be potentially manipulated using probiotics and antibiotics. Although our understanding of lung microbiome is rapidly improving, there is not enough studies that have looked at the standard technology and analysis, and many questions remain to be addressed.
